# Effects of saccharin on insulin sensitivity in adult, overweight individuals without diabetes: a real-world pilot study

**DOI:** 10.1210/jendso/bvag096

**Published:** 2026-04-17

**Authors:** Kenny Kalin, Karin Rådholm, Lisa Olsson, Valentina Tremaroli, Mark Woodward, Maria Wennberg, Fredrik Bäckhed, Olov Rolandsson

**Affiliations:** Department of Public Health and Clinical Medicine, Family Medicine, Umeå University, Umeå SE-907 87, Sweden; Primary Health Care Center Kärna and Department of Health, Medicine and Caring Sciences, Linköping University, SE-58183 Linköping, Sweden; The George Institute for Global Health, University of New South Wales, Barangaroo, NSW 2000, Australia; The Wallenberg Laboratory, Department of Molecular and Clinical Medicine, Institute of Medicine, Sahlgrenska Academy, University of Gothenburg, Sahlgrenska Universitetssjukhuset, Göteborg SE-413 45, Sweden; The Wallenberg Laboratory, Department of Molecular and Clinical Medicine, Institute of Medicine, Sahlgrenska Academy, University of Gothenburg, Sahlgrenska Universitetssjukhuset, Göteborg SE-413 45, Sweden; The George Institute for Global Health, University of New South Wales, Barangaroo, NSW 2000, Australia; The George Institute for Global Health, School of Public Health, Imperial College London, London W12 7RZ, UK; Department of Public Health and Clinical Medicine, Umeå University, Umeå SE-901 87, Sweden; The Wallenberg Laboratory, Department of Molecular and Clinical Medicine, Institute of Medicine, Sahlgrenska Academy, University of Gothenburg, Sahlgrenska Universitetssjukhuset, Göteborg SE-413 45, Sweden; Department of Clinical Physiology, Sahlgrenska University Hospital, Gothenburg SE-413 45, Sweden; Department of Public Health and Clinical Medicine, Family Medicine, Umeå University, Umeå SE-907 87, Sweden

**Keywords:** saccharin, insulin resistance, gastrointestinal microbiome, humans, overweight, sweetening agents

## Abstract

**Context:**

It has been suggested that consumption of saccharin, a widely used artificial sweetener, decreases insulin sensitivity in rodents and humans, but studies show conflicting results.

**Objective:**

To investigate if saccharin affects insulin sensitivity in a proof-of-concept study in humans using hyperinsulinemic-euglycemic clamp.

**Methods:**

In an open-label pilot study, we recruited 14 overweight participants without diabetes who were mean 60.5 (SD 4.1) years of age and had a body mass index of 27.6 (SD 0.7). Insulin sensitivity, assessed by hyperinsulinemic-euglycemic clamp, was determined before and after consumption of 5 mg/kg saccharin/day for 3 months. Blood was collected for analysis of diabetes-related biomarkers. Stool samples were collected before, during, and after saccharin consumption for microbiota profiling by 16S rRNA gene sequencing.

**Results:**

Thirteen of the 14 participants (6 men, 7 women) completed the study. There was no change in insulin sensitivity (mean M value difference [ΔM] −0.1, *P* = .85) or body weight (mean difference −0.1 kg, *P* = .70) after consumption of saccharin. However, the mean glycated hemoglobin decreased from 38.7 mmol/mol (SD 3.0) at visit 1 to 36.8 (SD 3.4) at visit 4 (*P* = .003). Overall, there was no change in composition or richness of the gut microbiota at the end of the study.

**Conclusion:**

This study did not demonstrate an association between saccharin intake and impaired insulin sensitivity in adult, overweight participants without diabetes assessed by hyperinsulinemic-euglycemic clamp.

Artificial sweeteners offer consumers an energy-neutral sweetener option. The consumption of artificial sweeteners has increased considerably in recent decades [1], even though this incline now seems to have slowed down [2]. The benefits of artificial sweeteners have, however, been questioned; they have been associated with weight gain [3], increased waist circumference [4], as well as increased risk of both cancer [5] and type 2 diabetes [6]. However, such observations have not been made in other studies [7].

Artificial sweeteners are heterogenous molecules that have contrasting human metabolic effects, including reduced glucose tolerance [8]. Glucose tolerance is a composite of insulin sensitivity, intestinal glucose uptake, glucose-dependent glucose disposal, endogenous insulin production, and incretin effects, resulting in systemic glucose homeostasis [9, 10]. The hyperinsulinemic-euglycemic clamp suppresses endogenous glucose production, which enables estimation of insulin sensitivity [11]; it is considered the gold standard to assess insulin sensitivity and calculate insulin resistance, which is the leading mechanism for the development of reduced glucose tolerance [9].

The mechanisms for how artificial sweeteners could modulate glucose tolerance have remained elusive. The gut microbiota plays a role in regulating a number of physiological processes [12], and alterations to its composition have been associated with cardiometabolic diseases, including type 2 diabetes [13]. Previous studies have found that a subset of artificial sweeteners, saccharin and sucralose, may impede glycemic control through alterations of the human gut microbiota [14, 15]. Conflicting results have, however, been found regarding saccharin's effect on glucose tolerance and gut microbiota composition. Studies by Suez et al [14, 15] have shown associations between short-term saccharin consumption, impaired glucose tolerance, and changes in gut microbiota composition, whereas a similar study by Serrano et al [16] did not find these associations. In light of the different results from previous studies using oral glucose tolerance tests, we assessed insulin sensitivity through the hyperinsulinemic-euglycemic clamp technique. In addition to hyperinsulinemic-euglycemic clamps, metabolism-related biomarkers in the blood as well as gut microbiota were analyzed in overweight participants without type 2 diabetes.

## Materials and methods

### Study population

This was an open-label experimental pilot study conducted in 2016. We recruited overweight participants who did not have diabetes. Exclusion criteria were a diagnosis of diabetes, treatment with antibiotics 3 months before inclusion and during the trial period, and bowel disease or medication known to affect glucose metabolism. We aimed to recruit 16 participants (8 women and 8 men) by advertising in local newspapers and managed to include 14 individuals, of whom 13 completed (7 women and 6 men) the study. We did not perform a power calculation because this was a pilot study, but aimed to include twice as many participants as in Suez et al, where 7 participants were included [14].

### Ethical considerations

All participants gave informed written consent before inclusion and the study was approved by the Regional Ethical Review Board in Umeå (Dnr 2015-244-31).

### Procedures

**Figure 1:**
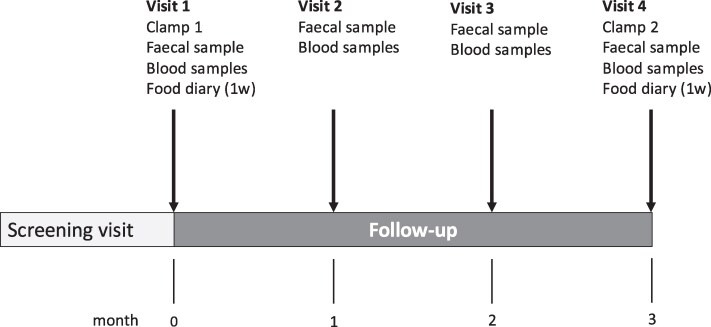
Study visit flow chart.

The design of the study is depicted in Fig. 1. After inclusion, the participants made 4 clinic visits. At study visit 1, height (to the nearest centimeter), weight (to the nearest 0.1 kg), and waist circumference (centimeter) were measured with the participants wearing light indoor clothing. Blood pressure was measured after a 10-minute rest in a sitting position. Fasting plasma glucose (HemocCue, Radiometer Medical Aps, Brønshøj, Denmark), glycated hemoglobin (HbA1c; TOSOH G5, Tosoh, Tokyo, Japan), and plasma C-peptide (Roche Diagnostics, Basel, Switzerland) were measured after a 10-hour overnight fast. All these measurements, except height, were recorded at all visits, including collection of fecal samples. Before visits 1 and 4, the participants registered their daily food intake for a week using food diaries. After visit 1, the participants were instructed to consume saccharin (Hermesetas) 5 mg/kg of body weight daily for 3 months, the maximum recommended dose by the European Food Safety Authority [17]. At each follow-up visit, the remaining saccharin tablets were counted to measure adherence.

### Measurement of insulin resistance

A hyperinsulinemic-euglycemic clamp [11] was used to evaluate insulin resistance at visit 1 and after 3 months (Fig. 1). Participants fasted overnight, and insulin was infused in 1 of 2 venous catheters while the participant was in resting in bed with the right hand in a hand incubator set at 37° C. A 20% glucose solution was infused in the other catheter. After a 10-minute insulin ladder period, a maintenance dose was administered for 120 minutes based on insulin 56 mU/m^2^ of body surface per minute. Plasma glucose level was clamped at 5.6 (±0.2) mmol/L Infusion of glucose was adjusted every 5 minutes. The insulin sensitivity at whole-body glucose metabolism at a steady state (M-value) was calculated by dividing the glucose infusion rate during the final 60 minutes of the clamp by body weight (mg/kg/min) [18]. A clinically significant increase or decrease of the insulin sensitivity has been suggested to an M-value difference (ΔM) of 15% or more between measurements [19].

The Homeostatic Model Assessment (HOMA) was used to indirectly estimate insulin resistance (HOMA-IR) and β-cell function (HOMA-%B), respectively, for each visit using measurements of fasting glucose and fasting c-peptide. HOMA calculations were performed using the HOMA2 calculator [20].

### Microbiota analyses

Total fecal genomic DNA was extracted using a repeated bead-beating method and amplified in duplicate reactions as previously described [21] using the dual-indexed primers 515F and 806R [22] targeting the V4 region of the 16S rRNA gene. Amplicons were sequenced in an Illumina Miseq instrument using the V2 kit (2 × 250-bp paired-end reads).

Illumina reads were merged and quality filtered using Usearch v.11 64-bit [23]. High-quality reads were processed to zero-radius operational taxonomic units (Zotus) by compiling the sequences into sets of unique reads and performing error correction using the UNOISE3 algorithm. The Zotus were assigned taxonomy using DADA2 [24] assignTaxonomy (minBoot = 50) and assignSpecies, using a formatted version of the Silva database (v.132) [25]. A phylogenetic tree of the sequences was created using MAFFT v.7.407 [26] and FastTree v.2.1.10 [27]. Zotus contributing less than 0.0015% to the total amount of reads were removed and analyses were performed on 1241 Zotus for the 52 samples from the 13 individuals who completed the study. Graphical representations and statistical analyses of gut microbiota profiles were performed using R v.3.5.1 with packages phyloseq v.1.26 [28] and ggplot2 v.3. For alpha-diversity, Faith's Phylogenetic Diversity was calculated using the package Picante (v1.7) [29]. β-diversity was analyzed using the Bray-Curtis dissimilarity index calculated using the package vegan v.2.5-4 [30] and visualized by principal coordinates analysis.

### Statistical methods

Categorical data are presented as numbers and corresponding proportions, whereas continuous data are expressed as mean values and SDs. Changes in insulin sensitivity (ΔM) and HbA1c were examined using linear mixed models where ΔM was adjusted for HbA1c and HbA1c was adjusted for body mass index (BMI). Unadjusted linear mixed models were used to assess changes in weight, BMI, waist circumference, systolic blood pressure, diastolic blood pressure, fasting glucose, C-peptide, HOMA-IR, and HOMA-%B across the 4 measurement time points. A *P* value < .05 was considered significant. Statistical analyses were performed using IBM SPSS statistics v28.0.1.0. For gut microbiota analyses, differences in phylogenetic diversity were assessed using linear mixed models across the 4 time points. Differential abundance of Zotus was assessed using the Wilcoxon signed-rank test to compare abundances at the end of the study compared to baseline; the Benjamini-Hochberg procedure was used to adjust for false discovery rate [31]. Significance was defined for features with an adjusted *P* <.05. Differences in overall composition were assessed based on the Bray-Curtis dissimilarity index using the adonis function [32, 33] in the vegan package v.2.5-4 [30].

## Results

### Participant characteristics

We recruited 14 overweight but not obese participants between 55 and 65 years of age with a BMI of 26 to 29 kg/m^2^, of whom 13 completed the study (Table 1). Participants had normotensive blood pressure, and glycemic control and β-cell function were in the normal ranges. Glycemic control, measured by HbA1c, improved over the study period (Table 1). However, there were no changes in fP-glucose or β-cell function, measured by HOMA-%B, and fP c-peptide (Table 1). No noteworthy changes in food intake were observed in the food diaries of participants who reduced their HbA1c during the study period; data not presented.

**Table 1:** Characteristics at baseline and follow-up measurements of 14 included participants

	Visit 1, n = 14	Visit 2, n = 14	Visit 3, n = 13	Visit 4, n = 13	*P*-value
Sex (men/women)	6/8	6/8	6/7	6/7	
Age (y)	60.5 (4.1)				
Weight (kg)	81.8 (7.9)	82.0 (7.9)	81.7 (7.9)	81.7 (7.9)	.70
BMI (kg/m^2^)	27.6 (0.7)	27.7 (1.1)	27.6 (1.1)	27.7 (1.1)	.23
WC (cm)	97.4 (7.5)	96.6 (5.6)	97.4 (6.4)	96.7 (6.0)	.79
SBP (mm Hg)	136.6 (15.7)	133.6 (19.1)	131.3 (12.7)	136.8 (13.1)	.07
DBP (mm Hg)	80.1 (6.7)	82.1 (10.5)	83.5 (8.2)	79.9 (7.9)	.**01**
HbA1c (mmol/mol)	38.7 (3.0)	37.7 (3.0)	37.1 (3.0)	36.8 (3.4)	.**003**
fP c-peptide (nmol/L)	0.79 (0.4)	0.82 (0.4)	0.84 (0.4)	0.81 (0.4)	.71
fP-glucose (mmol/L)	5.9 (0.7)	5.8 (0.7)	5.8 (0.7)	5.7 (0.4)	.70
HOMA-IR	1.8 (0.7)	1.9 (0.4)	1.9 (0.7)	1.9 (0.4)	.60
HOMA-%B	104.2 (21.7)	113.9 (30.7)	108.4 (22.8)	111.0 (22.4)	.62
M-value (mg/kg/min)	7.9 (1.5)	—	—	7.8 (1.5)	.85

All values except sex are mean and standard deviation (SD).

Abbreviations: BMI, body mass index; fP-glucose, fasting plasma glucose; fP c-peptide, fasting plasma c-peptide; HOMA-%B, homeostatic model assessment of β-cell function; HOMA-IR, homeostatic model assessment of insulin resistance; M-value, glucose metabolized; WC, waist circumference.

There were no changes in weight, waist circumference, or systolic blood pressure over the study period (Table 1).

### Insulin sensitivity

**Figure 2:**
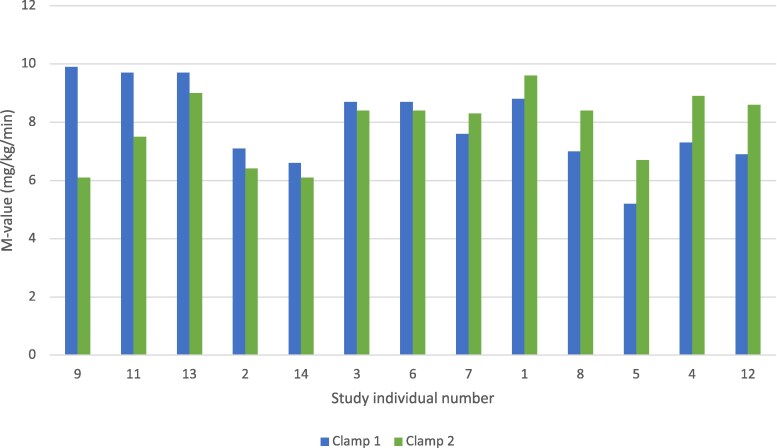
Individual changes in peripheral insulin sensitivity (M values) in nondiabetic, overweight individuals (n = 13) before and after saccharin intake for 3 months.

There was no change in insulin sensitivity from baseline to follow-up, measured by M-value and HOMA-IR (Table 1). The mean M-value difference between visit 1 and visit 4, ΔM, for all participants was −0.1 (*P* = .85). ΔM-change was not associated with HbA1c (*P* = .64). Individual M values from both insulin clamps are shown in Fig. 2. Two participants (9 and 11) had a reduction of clamp estimated insulin sensitivity of >15% at visit 4 with an individual reduction in M-value of 38.4% and 22.7%, respectively. These participants increased HOMA-IR and HOMA-%B during the study period. Participant 9 increased HOMA-IR from 1.81 to 2.28 and HOMA-%B from 121.4 to 161.5. Participant 11 increased HOMA-IR from 0.9 to 1.22 and HOMA-%B from 71.5 to 92.3.

### Gut microbiota composition

**Figure 3:**
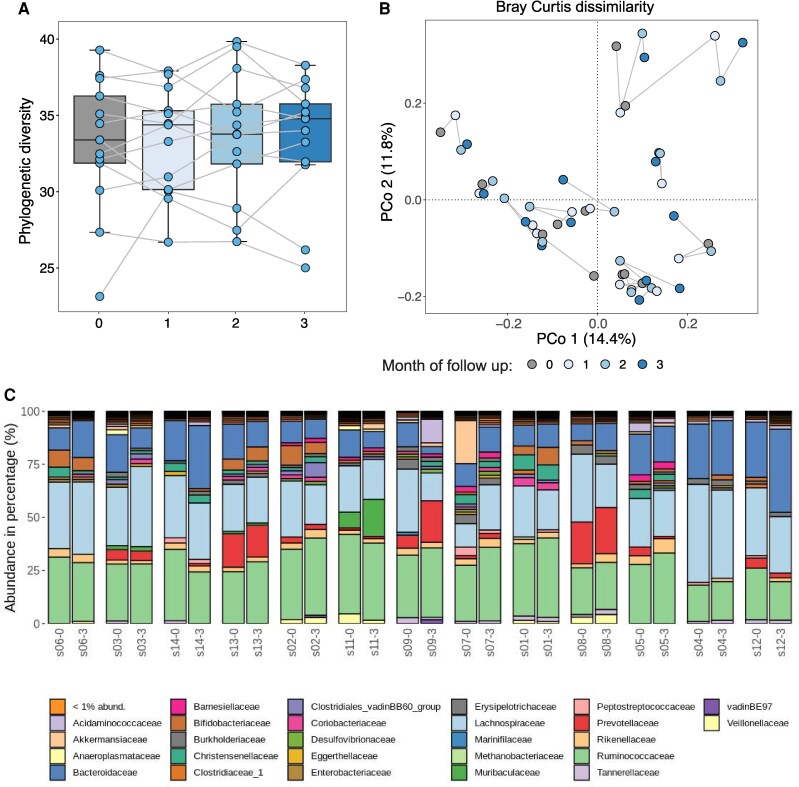
Gut microbiota profiles during saccharin intake. (A) Box plots showing phylogenetic diversity as a measure of species diversity (α diversity) during the intake of saccharin; the plots present phylogenetic diversity at baseline and each month during the study. Samples belonging to the same individual are connected by a gray line. (B) Principal coordinates analysis (PCoA) using the Bray-Curtis dissimilarity index evaluated the change of overall gut microbiota composition during the study. Samples belonging to the same individual are connected by a gray line. (C) Bar plots for the relative abundance of Zotus presented at the family level. Adjacent plots show abundance of families at baseline (0) and at the end of the study (3) for each individual; families present at a relative abundance lower than 1% are grouped into 1 category.

Fecal microbiota composition was profiled in the 52 samples from the 13 participants who completed the study, at visit 1 (baseline) and each month during the study (visits 2, 3, and 4). The analyses showed no significant change of α-diversity measured by phylogenetic diversity at the end of study compared to baseline (*P* = .92, Fig. 3A), and no shift in overall gut microbiota composition based on the Bray-Curtis dissimilarity index (adonis [30], 9999 permutations, *R*^2^ = 0.082, *P* = .98, Fig. 3B). We did not observe significant changes in the relative abundance of taxa at the end of the study compared to baseline at either Zotu, Table S1 [34] or family levels, Table S2 [34], (Fig. 3C).

## Discussion

In this study, we evaluated the impact of saccharin on insulin sensitivity using the hyperinsulinemic-euglycemic clamp, considered the gold standard for estimating insulin sensitivity [11]. We did not find any changes in insulin sensitivity after saccharin exposure. Additional assessments of HOMA-IR and HOMA-%B revealed no significant alterations except for 2 participants who demonstrated reduced insulin sensitivity at follow-up estimated by the M-value. Notably, these individuals also increased HOMA-IR and HOMA-%B during the study period.

The HbA1c reduction noticed in our study was not associated with a decrease in body weight. This might be an effect of caloric compensation where participants replaced carbohydrate intake with other energy sources. However, the food diary records did not reveal significant changes in dietary patterns among HbA1c reducers or participants that reduced M-value >15%, which was deemed a true M-value change, not resulting from intra-participant test result variability [19]. Alternatively, the reduction in HbA1c might have been a consequence of increased physical activity, which was not monitored in the study.

Previous studies have indicated that artificial sweeteners such as sucralose, aspartame, and saccharin can affect human glycemic control [14, 15, 35], whereas for other sweeteners no effect has been observed [36]. However, to our knowledge, no study has specifically estimated saccharin's potential effect on insulin sensitivity using gold standard methodology in nondiabetic, overweight individuals with saccharin consumption maintained at maximum Acceptable Daily Intake (ADI). Our results are in line with the findings of Serrano et al [16], who in a double-blind, randomized, placebo-controlled study included 46 healthy participants out of whom the saccharin intervention group comprised 13 individuals, with a mean age of 28.91 (±2.6 SEM) and a mean BMI of 22.4 (±0.53 SEM). The study showed that exposure to saccharin at maximum ADI for 2 weeks did not reduce glucose tolerance or affected concentrations of insulin, c-peptide, glucagon, or GLP-1. The authors commented that 1 possible reason for not finding an effect of saccharin on glucose tolerance was that the duration of the performed intervention was limited to 2 weeks. Because our results align with prior findings and furthermore are based on a 12-week intervention, our findings add to the body of evidence indicating that short-term saccharin consumption has limited impact on glucose tolerance. In contrast to our and Serrano et al's studies, Suez et al have reported that saccharin in short term studies affects glucose metabolism by reducing glucose tolerance [14, 15]. In 2014 Suez et al showed, in a group of 7 healthy volunteers, that a 1-week period of exposure to maximal daily intake of saccharin negatively affected glucose tolerance among a subset of the exposed participants [14]. In a more recent study, the authors observed a similar negative effect on glucose tolerance in humans, who had never previously been exposed to saccharin, after an exposure to 20% acceptable daily intake of saccharin for 2 weeks [15]. Both studies by Suez et al assessed glucose tolerance through changes in the incremental area under the glucose curve by repeated glucose tolerance testing. Suez et al hypothesized that the mechanism behind the observed negative effect on glucose tolerance is caused by a blunting of glucose-stimulated insulin secretion [15]. However, in contrast to this hypothesis, our study could not detect any changes in HOMA-%B after exposure to saccharin. As stated in the 2023 World Health Organization (WHO) meta-analysis concerning effects of consumption of artificial sweeteners [37], long-term studies of chronic consumption showed an increased risk of developing type 2 diabetes. Long-term studies were mainly observational and the association between artificial sweetener consumption and type 2 diabetes could be a result of reverse causation and/or residual confounding. Even though solid efforts have been made to adjust for potential reverse causation, the association remains. It would therefore be of value to perform a randomized controlled trial assessing effects on insulin sensitivity after a long-term saccharin intervention and analyze the data after stratification of BMI and saccharin.

There are substantial methodological differences between our, Serrano et al’s and Suez et al’s studies concerning the techniques used to assess glucose tolerance. In the studies by Serrano et al and Suez et al, an oral glucose tolerance test was used. This test can detect reduced glucose tolerance but not distinguish if the intolerance is caused by reduced insulin sensitivity or reduced β-cell function. Furthermore, in the 2022 study by Suez et al, glucose metabolism was assessed by repeated oral glucose tolerance tests with a 50-g glucose load, which contrasts the recommendations by the American Diabetes Association [38], which recommend a 75-g glucose load. Also, the oral glucose tolerance test in the study by Suez et al was administrated by the participants themselves, which might lead to an overestimation of the reduction of glucose tolerance.

In agreement with Serrano et al [16], but in contrast to Suez et al [14], we did not observe differences in gut microbiota before and after saccharin consumption using 16S rRNA gene profiling. In the 2022 study [15], Suez et al demonstrated that individuals with impaired glucose tolerance following saccharine exposure were associated with altered gut microbiome assessed by shotgun metagenomic sequencing. Our study was not sufficiently powered to analyze potential personalized effects of saccharin intake on insulin sensitivity, because only 2 participants demonstrated reduced insulin sensitivity. Accordingly, we cannot exclude that a subpopulation of individuals will develop insulin resistance following saccharine exposure.

The American Diabetes Association has concluded that artificial sweeteners appear to have limited influence on glycemic control, but state that individuals with type 2 diabetes may use artificial sweeteners to reduce caloric intake [39]. The 2023 WHO guidelines on nonsugar sweeteners [40] advise against their use for weight loss or reducing noncommunicable disease risk. However, this recommendation excludes individuals with existing diabetes, as the guideline's scope did not cover studies involving people with existing diabetes. Short-term as well as long-term studies show conflicting results as to the connection between artificial sweeteners and benefits of, or risk for noncommunicable disease. Short-term, mainly interventional studies show associations with improved metabolic markers [37] with some notable exceptions [14, 15]. Our findings add to the existing body of evidence suggesting that saccharin does not reduce insulin sensitivity in the short term.

A strength in our study is that we estimated insulin sensitivity with the gold standard hyperinsulinemic-euglycemic clamp technique. In addition, the participants consumed saccharin for 3 months at the maximum recommended dose, a setting in which saccharin should have affected insulin sensitivity, especially in individuals who are overweight. However, there are some limitations. The precision of glucose clamp measurements relies on the proper execution of the clamp, a task made challenging because of the technical aspects of the procedure. There are also limitations such as the open-label design and the relatively small sample size, which also prevents sufficient power to assess individual responses. Recruitment via local newspaper advertisements may have introduced selection bias and thereby limited the generalizability of our findings. Furthermore, because we did not exclude participants with previous consumption of artificial sweeteners, some participants’ microbiota may already have adapted to saccharin.

## Conclusion

In conclusion, our study did not demonstrate that saccharin impairs insulin sensitivity in overweight participants with a consumption at maximum ADI of saccharin for three months.

## Data Availability

Restrictions apply to the availability of some or all data generated or analyzed during this study to preserve patient confidentiality or because they were used under license. The corresponding author will on request detail the restrictions and any conditions under which access to some data may be provided. The sequencing data generated in this study have been deposited in the European Nucleotide Archive (ENA) at EMBL-EBI under accession number PRJEB108714 (https://www.ebi.ac.uk/ena/browser/view/PRJEB108714).

## Abbreviations

ADI: Acceptable Daily Intake
BMI: body mass index
HbA1c: glycated hemoglobin
HOMA-%B: homeostatic model assessment of β-cell function
HOMA-IR: homeostatic model assessment of insulin resistance
WHO: World Health Organization
Zotus: zero-radius operational taxonomic units
